# Chewie Nomenclature Server (chewie-NS): a deployable nomenclature server for easy sharing of core and whole genome MLST schemas

**DOI:** 10.1093/nar/gkaa889

**Published:** 2020-10-17

**Authors:** Rafael Mamede, Pedro Vila-Cerqueira, Mickael Silva, João A Carriço, Mário Ramirez

**Affiliations:** Instituto de Microbiologia and Instituto de Medicina Molecular João Lobo Antunes, Faculdade de Medicina, Universidade de Lisboa, Av. Professor Egas Moniz, 1649-028 Lisboa, Portugal; Instituto de Microbiologia and Instituto de Medicina Molecular João Lobo Antunes, Faculdade de Medicina, Universidade de Lisboa, Av. Professor Egas Moniz, 1649-028 Lisboa, Portugal; Instituto de Microbiologia and Instituto de Medicina Molecular João Lobo Antunes, Faculdade de Medicina, Universidade de Lisboa, Av. Professor Egas Moniz, 1649-028 Lisboa, Portugal; Instituto de Microbiologia and Instituto de Medicina Molecular João Lobo Antunes, Faculdade de Medicina, Universidade de Lisboa, Av. Professor Egas Moniz, 1649-028 Lisboa, Portugal; Instituto de Microbiologia and Instituto de Medicina Molecular João Lobo Antunes, Faculdade de Medicina, Universidade de Lisboa, Av. Professor Egas Moniz, 1649-028 Lisboa, Portugal

## Abstract

Chewie Nomenclature Server (chewie-NS, https://chewbbaca.online/) allows users to share genome-based gene-by-gene typing schemas and to maintain a common nomenclature, simplifying the comparison of results. The combination between local analyses and a public repository of allelic data strikes a balance between potential confidentiality issues and the need to compare results. The possibility of deploying private instances of chewie-NS facilitates the creation of nomenclature servers with a restricted user base to allow compliance with the strictest data policies. Chewie-NS allows users to easily share their own schemas and to explore publicly available schemas, including informative statistics on schemas and loci presented in interactive charts and tables. Users can retrieve all the information necessary to run a schema locally or all the alleles identified at a particular locus. The integration with the chewBBACA suite enables users to directly upload new schemas to chewie-NS, download existing schemas and synchronize local and remote schemas from chewBBACA command line version, allowing an easier integration into high-throughput analysis pipelines. The same REST API linking chewie-NS and the chewBBACA suite supports the interaction of other interfaces or pipelines with the databases available at chewie-NS, facilitating the reusability of the stored data.

## INTRODUCTION

The importance of distinguishing strains within the same microbial species has been proven critical for identifying chains of transmission and understanding pathogen evolution, as recently illustrated by the SARS-CoV-2 pandemic (1,2). The advent and widespread adoption of high-throughput sequencing allowed leveraging genomic information for this purpose (1,2). One of the most common approaches in bacterial typing is gene-by-gene methods, which extend the concept of multilocus sequence typing (MLST) to include all genes present in the core genome of a given species (cgMLST) or, trying to cover a significant fraction of a species’ pan-genome, in whole genome (wgMLST) (3). Current software approaches implementing these wg/cgMLST typing methods suffer from standardization issues when comparing results between different tools and between different laboratories or users (4).

We have previously developed a suite, chewBBACA (5), allowing the creation of gene-by-gene schemas and performing allele calls on assembled draft genomes. Since chewBBACA was designed to perform local analysis to address concerns over data privacy and scalability, it has the drawback that small adjustments in parameters may lead to inconsistencies between runs. Moreover, the software allows users to create their own wg/cgMLST schemas but currently no tool is available for the easy sharing of schemas, which potentially hampers long-term and multinational studies, as well as the reusability of already published schemas (6,7).

There are well-established websites for performing gene-by-gene analyses, such as PubMLST (https://pubmlst.org/) (8) and EnteroBase (https://enterobase.warwick.ac.uk/) (9), that centralize analysis and hosting of public and private schemas. chewBBACA does not depend on a web server and by enabling local analyses and schema creation allows for scalable and private analyses of genomes, but the existing implementation lacked an easy way to share schemas and the associated allelic information, which is possible in a centralized solution.

In order to allow users to share gene-by-gene typing schemas and for a common allelic nomenclature to be maintained (10), we developed chewie-NS, a nomenclature server based on the TypOn ontology (11) offering a web interface that also integrates directly with local instances of chewBBACA and can be programmatically accessed by external resources. Chewie-NS aims to complement the private local analysis of strains by also allowing the simple communication of results while providing an interface for users to easily explore the allelic diversity within species. The importance of the latter is becoming increasingly clear with the recognition that bacterial phenotypes can be profoundly altered by allelic variants (12,13). Other publicly available web services require submission of raw data, something that may raise privacy and ownership concerns, while our approach of enabling local analyses is more flexible and scalable, and respects data privacy concerns. Current wg/cgMLST typing methods suffer from standardization difficulties or issues that manifest not only when trying to reconcile results from different tools, but also when the same tool is run at different times with small adjustments in parameter values or database modifications that may lead to inconsistencies. This is an even more complex problem than it was for classical MLST methods (14). Having a repository of schemas, their associated parameters and the allelic diversity identified will allow the consistent use of gene-by-gene typing schemas by different groups and to build upon the results of different studies to monitor microbial populations and study outbreaks.

Chewie-NS is available at https://chewbbaca.online and its source code is available at https://github.com/B-UMMI/Chewie-NS. Detailed documentation, including a descriptive tutorial on how to deploy and use the server, can be found at https://chewie-ns.readthedocs.io. Additionally, a tutorial version of the server aiming at familiarizing users with the integration between the chewBBACA suite and chewie-NS, which allows users to perform mock submissions of schemas and synchronizations without the need to register and with a much reduced database, is available at https://tutorial.chewbbaca.online/.

## DATABASE CREATION

### Backend

**Figure 1.**
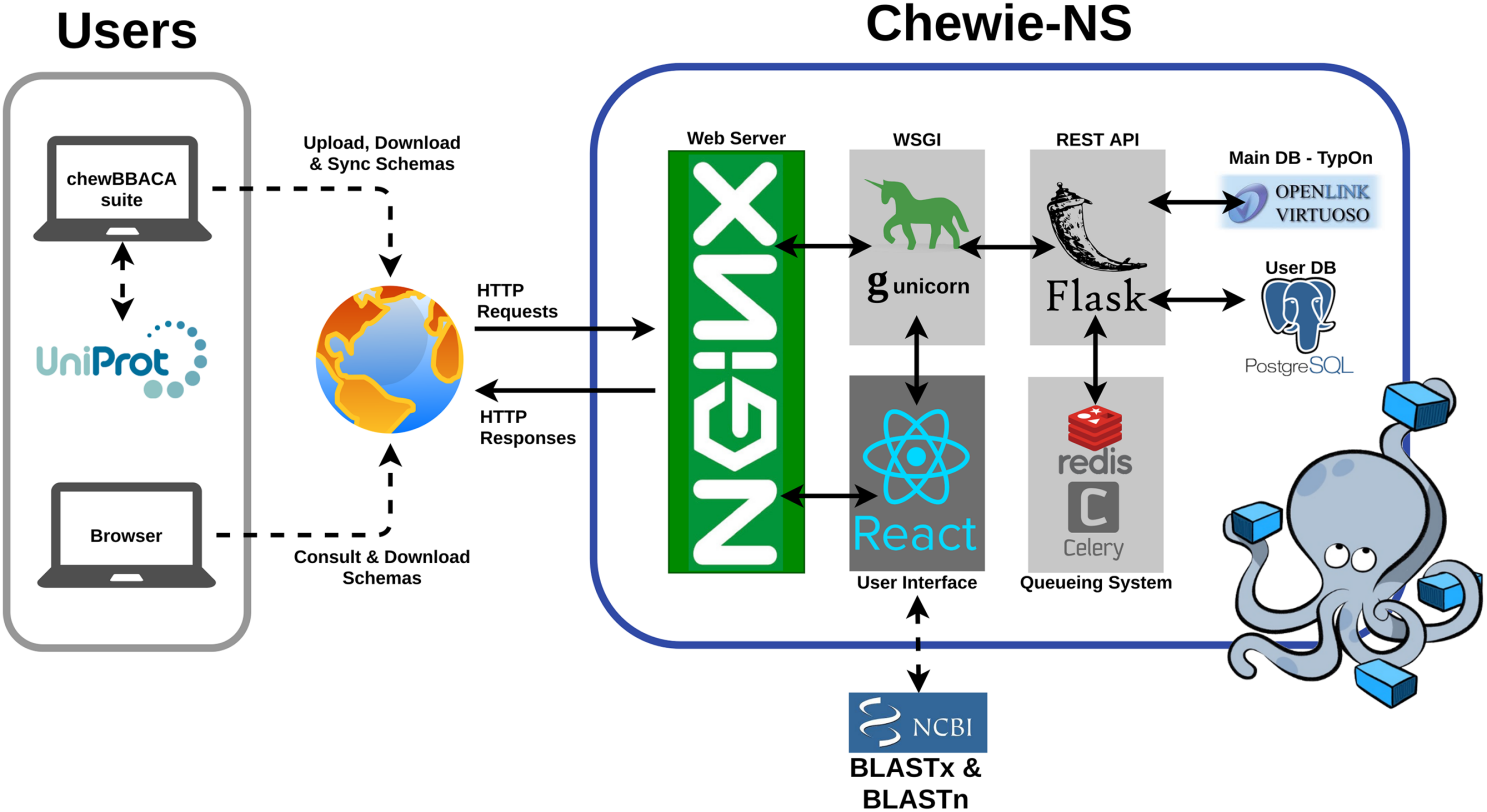
The chewie-NS service: global overview of the technologies used and API connectivity.

The architecture of chewie-NS is shown schematically in Figure 1. The backend component of chewie-NS makes use of the Virtuoso triple store (v. 7.2.6) (https://virtuoso.openlinksw.com/). This database management system allows the integration of a Resource Description Framework to implement the TypOn ontology (11) structure to store schema data. Additionally, a PostgreSQL database (v. 10) (https://www.postgresql.org/) was adopted for user management. These databases are accessible through a Python 3 REST API developed in the Flask (v. 1.1.0) (https://flask.palletsprojects.com/en/1.1.x/) web development microframework, which allows requests through defined endpoints and facilitates programmatic access to the nomenclature server. Requests and HTTPS connections are handled by a web server, NGINX (v. 1.17) (https://www.nginx.com/), that communicates with Gunicorn (v. 20.0.4) (https://gunicorn.org/), a WSGI application server capable of running multiple processes of the web application and distributing incoming requests to ensure scalability and load balancing. A queueing system was implemented to manage all tasks with possible concurrent user access through Redis (v. 5.0.6) (https://redis.io/) and Celery (v. 4.4.0rc2) (https://docs.celeryproject.org/en/stable/getting-started/introduction.html).

### Frontend

The user interface (UI) for chewie-NS was built with the JavaScript frameworks React (v. 16.12.0) (https://reactjs.org/) and Material-UI (v. 4.9.14) (https://material-ui.com/). The UI provides a list of available schemas and displays relevant schema and locus statistics in a responsive and interactive manner. Access to daily updated compressed files of the schemas for download and local use is also provided. All interactive charts were rendered with the graph visualization library Plotly.js (v. 1.52.1) (https://plotly.com/javascript/) through its React component, react-plotly (v. 2.4.0) (https://plotly.com/javascript/react/).

### Chewie-NS usage

#### Local installation

Deployment of local instances can be easily achieved through Docker Compose (https://www.docker.com/) (available at https://github.com/B-UMMI/Chewie-NS). The use of a container orchestrator (https://docs.docker.com/compose/) supports the easy deployment of local instances independently of the hardware available, allowing the creation of private trusted databases if public access is not possible. Instructions on how to achieve this can be found at https://github.com/B-UMMI/Chewie-NS. This can be particularly important for national public health institutions in the context of restrictive or ambiguous data sharing laws because it allows stricter user access control.

#### Application programming interface

A RESTful API also referred to as a RESTful web service or REST API, i.e. based on representational state transfer (REST), is available. The user can interact with chewie-NS’s API through the web interface, by clicking on the ‘API’ button on the menu. This will open a page with Swagger UI (https://swagger.io/tools/swagger-ui/), a user-friendly tool for the user to interact directly with the REST API. Programmatic access is also possible through command line applications such as curl or tools such as Postman (https://www.postman.com/). Chewie-NS’s REST API allows interaction with the PostgreSQL database to manage user registrations on local instances. Through the API, users are also able to query the Virtuoso database to download compressed schemas, search for specific alleles and query data about specific species, loci or alleles.

### Web interface

#### Schemas overview

**Figure 2.**
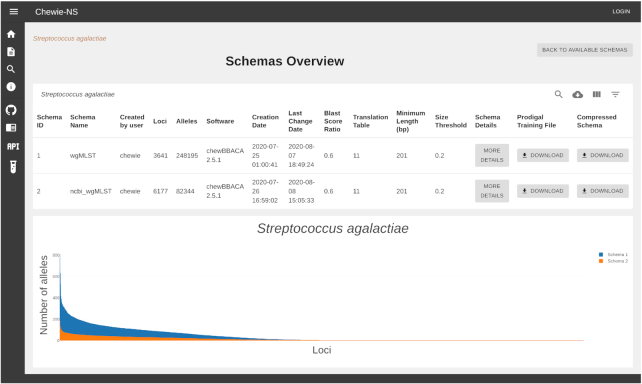
The schemas overview page of chewie-NS.

A table summarizes the species and number of schemas available for each species in chewie-NS. Selecting a species leads to another table (Figure 2) with a list of relevant information about each available schema, namely the schema internal identifier, the user provided schema name, the username of the creator, the number of loci in the schema, the number of alleles, the software and version used to create the schema, the date of creation, the date of the last modification, the BLAST (15) score ratio selected, the translation table used, the minimum locus length and size threshold. In the table, there is a link to download the compressed file of the schema and the training file used to create it, both necessary to use the schema locally with the chewBBACA suite. Each table entry has also a link to a page containing more details about the schema. Below this table, an interactive bar chart displays the number of alleles per locus for each schema. The user can zoom in on the chart to obtain a better view of a given set of loci and can click on a bar to go to a page with more details on that particular locus.

#### Schema details

**Figure 3.**
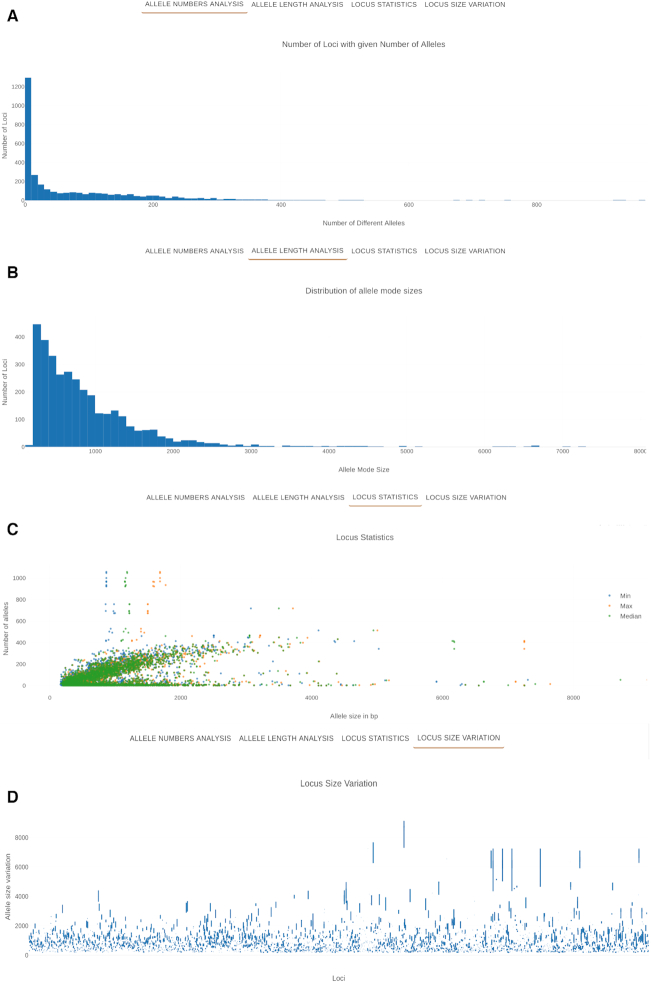
Summary charts displaying relevant information on a given schema. (**A**) Distribution of loci by number of alleles. (**B**) Distribution of loci by allele mode size. (**C**) Representation of summary statistics (minimum allele size in blue, maximum allele size in orange and median allele size in green) for each locus. (**D**) Box plots of loci size distribution; the loci in the *x*-axis are ordered by locus ID.

**Figure 4.**
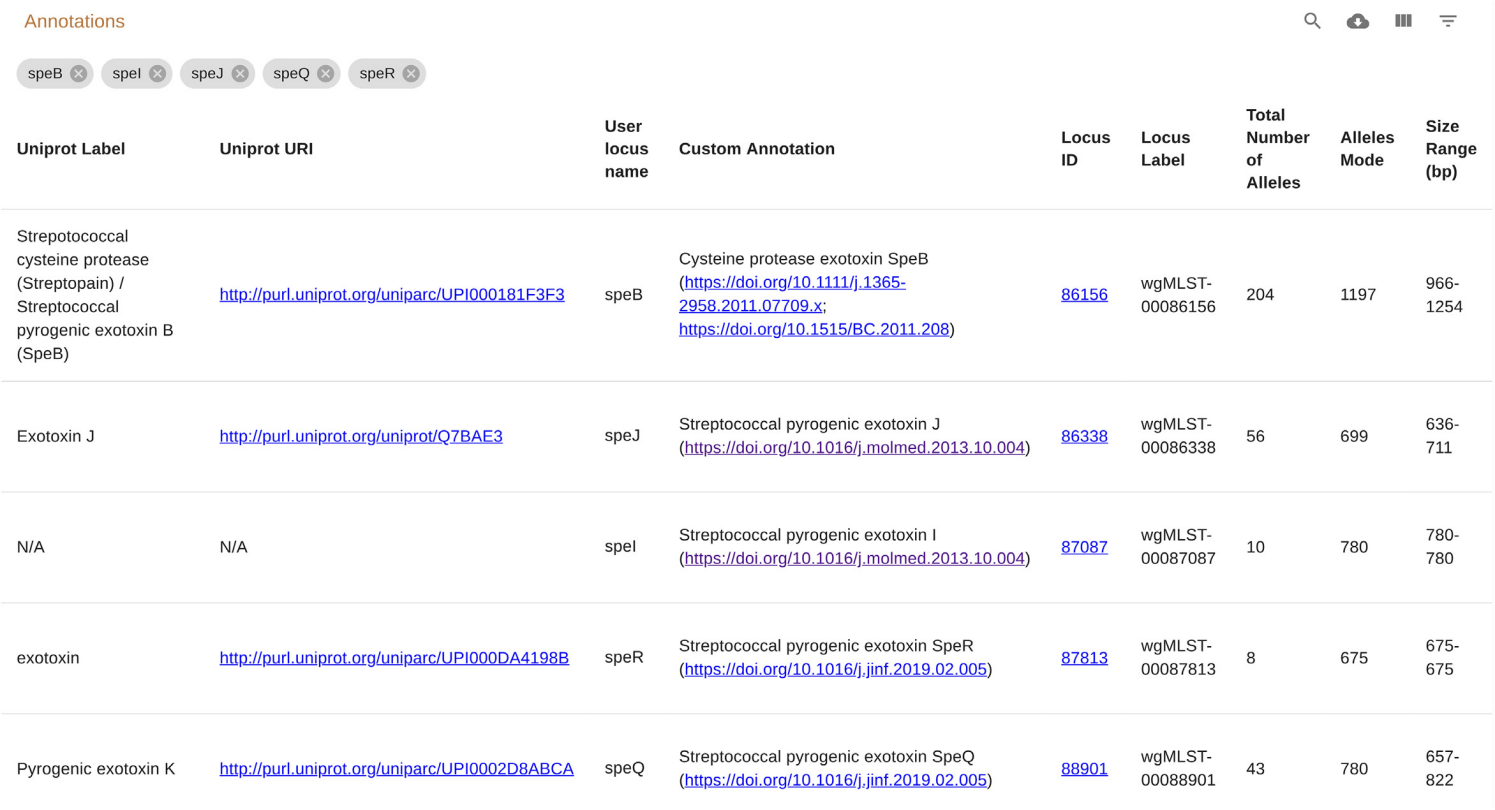
Schema loci table: search functionality. The figure presents the results of filtering for *speB, speI, speJ, speQ* and *speR* in the *user locus name* field of the *Streptococcus pyogenes* schema 1. Note that the user provided annotations complement and correct some of the annotations retrieved from UniProt.

The schema evaluation and annotation page contains a description of the schema provided by the schema creator. During the schema upload, this information can be provided in a file using markdown, a simple plain-text-formatting syntax that allows the easy integration of hyperlinks, tables and images, allowing for a rich use of data for the description of the schema. Below this table are four charts in different tabs. Two charts (Figure 3) display characteristics of the schema: the distribution of the number of alleles per locus and of locus size. Two interactive charts represent for each locus its size summary statistics versus the number of alleles, and another a box plot of the size distribution of each locus. In all charts, the user can zoom in on particular regions for more detailed inspection and, on the latter two, clicking on the chart element opens a page with more information on that particular locus. Below the charts is a table of all the loci in the schema, including relevant information for each locus. This table, as all other tables of chewie-NS, is searchable, facilitating finding loci with particular characteristics (Figure 4). Similarly to other tables, the table can also be exported in comma-separated values format.

#### Locus details

Already in the schema evaluation and annotation page is shown most of the information of each locus. This includes the internal locus identification and label, the automated annotation created by chewBBACA including a link to the relevant UniProt (13) page, a user locus name and user custom annotation (supporting markdown syntax), number of alleles and allele size information. The possibility of schema creators offering their own annotation allows for domain-specific information to be added to the schema, including potentially richer complementary data and links to relevant external resources. Two charts are offered, one summarizing the size distribution of the alleles (frequency of binned sizes) and the other representing the sizes of each allele. Direct links to perform BLAST searches using allele 1 of that locus (BLASTn and BLASTx) are available at the bottom of the page, allowing the user to check for similarities that could offer further insights into the origin or likely function of the protein potentially encoded by the locus. A multi-fasta file containing the alleles of the locus can be downloaded by the users from this page. On a different page, a simple query feature allows finding exact matches to specific sequences stored in any of chewie-NS’s databases, returning the loci and schemas where the sequence is found.

#### Integration with the chewBBACA suite and use of the API

By taking advantage of chewie-NS’s API, chewBBACA is capable of handling not only the schema creation, but also its upload, synchronization and download. Users of chewBBACA registered in chewie-NS and with contributor privileges will be able to automatically upload a novel schema, making it available in chewie-NS. Any authorized registered user can also contribute novel alleles identified in local analyses to chewie-NS, contributing to the incremental development of the schema. This involves only allele information without the need to share a complete allelic profile with chewie-NS. On the other hand, one does not have to be registered to download any of the data stored in chewie-NS, including the compressed schemas or the novel alleles submitted to the chewie-NS database and that were not present on the compressed file to update the local schema. Detailed instructions on the chewBBACA commands to achieve this can be found at https://chewie-ns.readthedocs.io/en/latest/user/chewbbaca.html.

If a user creates a schema through a software other than chewBBACA, the API can still be used to submit this new schema to chewie-NS or to add novel alleles to non-chewBBACA schemas. A user would have to register with chewie-NS and would have to take on the responsibility for making the correct API calls for schema submission. Furthermore, it would be up to each user submitting new alleles to guarantee the consistency of these novel alleles with the schema originally deposited. These functions are handled transparently by chewBBACA in its interaction with chewie-NS. Anyone can use the API to download any of the schemas deposited in chewie-NS to be run locally with chewBBACA or any other allele-calling algorithm. These multiple ways in which a user can interact with chewie-NS allows tailoring the sharing of information to user preferences or to restrictions imposed on particular users.

In order to facilitate familiarization of the interaction between chewBBACA and chewie-NS, a tutorial website was created (https://tutorial.chewbbaca.online/), together with step-by-step instructions on how to perform mock operations with a small size schema (https://chewie-ns.readthedocs.io/en/latest/user/tutorial.html). This allows users to perform submissions and synchronization of schemas without the need for registering. The schemas submitted to the tutorial site are not permanent and are removed automatically 48 h after creation.

For reproducibility and traceability purposes, a feature to retrieve database snapshots at specific dates is available, allowing a user to be able to recover the exact schema, as it was available on a given date. Full documentation of the schemas allows for traceability, which is critical in public health applications. These various options will continue to allow data privacy, while striving for a common nomenclature. The detailed parameterization associated with each schema created with chewBBACA and the consistency checks implemented in chewie-NS mean that no human curation is necessary after the schema creation step, contributing to the rapid update of the database and exchange of information. However, although chewie-NS can be used to store and retrieve information of schemas not created with chewBBACA, it does not currently automatically guarantee the consistency of newly submitted alleles since each allele-calling algorithm will have specific parametrization requirements. Nevertheless, these can be implemented in the future as other allele-calling algorithms make use of the chewie-NS platform.

## DISCUSSION

Chewie-NS accomplishes four important goals. First, it stores all the information required to define a chewBBACA schema, facilitating accessibility of schemas so that different schemas can be easily compared and evaluated. Second, it maintains a public compendium of the variability in each locus. Since chewBBACA loci are open-reading frames (ORFs), this will allow monitoring the variability of the proteins potentially encoded by these loci. This is important when studying microbial pathogens because small variations in sequence can lead to dramatic changes in virulence (16) or antimicrobial resistance (12). On the other hand, allelic diversity can also be indicative of stabilizing or diversifying selective pressures, which in turn can be leveraged to obtain insights into pathogen evolution or interaction with the host (17). Allelic diversity is also important in reverse vaccinology (18) and to monitor the continued potential efficacy of some available vaccines (19). Third, through its integration with chewBBACA, it offers a simplified way for the user to control the flow of information between the local instance and chewie-NS. This is important to keep the local instance of chewBBACA updated with the current common nomenclature stored in chewie-NS databases and to contribute new alleles to the common databases, but it also allows for limited sharing of data to comply with any regulations the user may be operating under. Finally, the proposed workflow hopes to stimulate and facilitate data sharing between users using the same schemas, allowing for a faster detection of strain similarity, therefore contributing for genomic epidemiology studies and also faster outbreak detection and investigation by expediting strain comparison between different laboratories or institutions. Although the current integration of chewBBACA with chewie-NS facilitates the user interaction with chewie-NS when using this allele-calling software, the API can be exploited by other allele-calling software to also interact directly with chewie-NS, allowing an easier submission of schemas and of new alleles to schemas not created with chewBBACA.

The definition of a wg/cgMLST schema involves not only the choice of the target loci and of what constitutes a locus [for instance, an ORF as defined by Prodigal (20) in the case of chewBBACA, or a fragment of DNA between two primers in traditional MLST], but also the algorithm and parameters of the allele-calling software. If one uses the same set of target loci defined in the same way, but a different allele-calling software, one cannot guarantee that the alleles called for a given isolate would be the same as with another allele-calling software, a problem that can potentially become all the more acute with the identification of novel alleles. Even when using the same allele-calling software, if the parameters used are different, the alleles identified in a given isolate may also be different. Moreover, the addition of novel alleles to a schema that do not conform with the parameters defined initially may have hard to anticipate consequences on the subsequent allele-calling processes. Upstream of this, we would like to stress the importance of the use of shared assembly pipelines, to ensure that the deposited allele sequences are determined based on standardized procedures, as it has been shown that different assemblers can result in different variants in the assembly (21) and that this variability can introduce artificial allelic variability, even when using the same schema and allele-calling software.

The possibility of setting up local instances of chewie-NS in a simplified way using Docker Compose facilitates creating private services that can cater to trusted groups of users and allow the implementation of chewie-NS in institutions operating under strict privacy rules. In a public health context, this can also be used to deploy services allowing an easier communication between different agencies operating under distinct mandates.

The databases currently available in the public instance of chewie-NS (https://chewbbaca.online/) include schemas developed within the INNUENDO project (7) for *Salmonella enterica*, *Campylobacter jejuni*, *Escherichia coli* and *Yersinia enterocolitica*, a schema developed for *Arcobacter butzleri* (6), an adaptation of a schema generated using the Ridom SeqSphere+ software for *Acinetobacter baumannii* (22) and in-house developed schemas for *Streptococcus agalactiae* and *Streptococcus pyogenes*. However, we expect that users of chewBBACA and of other allele-calling software will increasingly contribute schemas for these as well as additional species to be deposited in chewie-NS.

## DATA AVAILABILITY

Chewie-NS is freely accessible at https://chewbbaca.online/. Its source code is hosted at https://github.com/B-UMMI/Chewie-NS together with instructions on how to deploy it locally using Docker Compose and the documentation can be found at https://chewie-ns.readthedocs.io/. A tutorial version of the server, which allows users to perform mock submissions of schemas and synchronizations with a much reduced database, can be accessed at https://tutorial.chewbbaca.online/.
